# Inhibition of UGT2B7 Enzyme Activity in Human and Rat Liver Microsomes by Herbal Constituents

**DOI:** 10.3390/molecules23102696

**Published:** 2018-10-19

**Authors:** Nurul Huda Abdullah, Sabariah Ismail

**Affiliations:** Centre for Drug Research, Universiti Sains Malaysia, Gelugor 11800, Pulau Pinang, Malaysia; nha.nurulhuda@gmail.com

**Keywords:** zidovudine, UGT2B7, andrographolide, mitragynine, zerumbone

## Abstract

The co-use of conventional drug and herbal medicines may lead to herb-drug interaction via modulation of drug-metabolizing enzymes (DMEs) by herbal constituents. UDP-glucuronosyltransferases (UGTs) catalyzing glucuronidation are the major metabolic enzymes of Phase II DMEs. The in vitro inhibitory effect of several herbal constituents on one of the most important UGT isoforms, UGT2B7, in human liver microsomes (HLM) and rat liver microsomes (RLM) was investigated. Zidovudine (ZDV) was used as the probe substrate to determine UGT2B7 activity. The intrinsic clearance (V_max_/K_m_) of ZDV in HLM is 1.65 µL/mg/min which is ten times greater than in RLM, which is 0.16 µL/mg/min. Andrographolide, kaempferol-3-rutinoside, mitragynine and zerumbone inhibited ZDV glucuronidation in HLM with IC_50_ values of 6.18 ± 1.27, 18.56 ± 8.62, 8.11 ± 4.48 and 4.57 ± 0.23 µM, respectively, hence, herb-drug interactions are possible if andrographolide, kaempferol-3-rutinoside, mitragynine and zerumbone are taken together with drugs that are highly metabolized by UGT2B7. Meanwhile, only mitragynine and zerumbone inhibited ZDV glucuronidation in RLM with IC_50_ values of 51.20 ± 5.95 μM and 8.14 ± 2.12 µM, respectively, indicating a difference between the human and rat microsomal model so caution must be exercised when extrapolating inhibitory metabolic data from rats to humans.

## 1. Introduction

Drug-metabolizing enzymes can be categorized into two groups which are Phase I and Phase II enzymes. A compound can be metabolized either during one of the phases or both to be excreted out from the body. Uridine 5′-diphospho-glucuronosyltransferases (UGTs) are the major Phase II metabolizing enzymes responsible for the reaction known as glucuronidation [1]. The glucuronidated metabolites have higher polarity and water solubility making them easily excreted by urine and bile. According to the review by Wells et al. [2] in humans approximately 40–70% of clinical drugs are metabolized through this pathway. Nevertheless, endobiotics such as bilirubin, steroids, retinoids and bile acids also undergo this route of detoxification [3].

UGT2B7 appears to be a very important human UGT isoform. Drugs that have been identified to be metabolized by this isoform are the opioids such as codeine, morphine and oxycodone [4,5], the non-steroidal anti-inflammatory drugs (NSAIDs) including diclofenac, niflumic acid and mefanamic acid [6], the anticonvulsant valproic acid [7] and zidovudine, the therapeutic agent used in HIV treatment [8]. Additionally, harmful non-drug xenobiotics such as 2-acetylaminofluorene and benzo[α]pyrene to which humans might be exposed through inhalation or dermal contact also undergo glucuronidation via UGT2B7 to be eliminated from the body [9].

Herbal medicines have been used to treat various ailments and diseases since millennia. Nowadays, herbal medicines are taken as complementary and/or alternative medicines (CAMs) by people around the world, especially by the patients with chronic diseases such as diabetes, cancer, and human immunodeficiency virus (HIV) [10,11,12]. Furthermore, a study indicates that CAM therapies are mostly taken as complementary medicines along with conventional medicines rather than as alternatives medicines [13]. Concurrent consumption of herbs and drugs raises the concern about interactions between herbs and drugs [14]. Herbal medicines contain numerous bioactive constituents which are responsible for their therapeutic effects. These herbal constituents might share the same metabolic pathway with the drug, therefore, inhibition of the drug-metabolizing enzyme activity may decrease the drug’s metabolic clearance and increase its level in the body which in turn may lead to toxicity. A recent study reported that sauchinone, an active principle of the herb *Saururus chinensis* elevated the zidovudine (ZDV) concentration in mice blood by inhibiting UGT2B7 activity [15]. This can cause haematological toxicity [16]. Another study revealed that curcumin from *Curcuma* species [17] inhibited UGT2B7 in vitro [18,19]. 

All this suggests that determination of the causative herbal constituent(s) of herb-drug interactions associated with UGT2B7 is required as many important drugs are metabolized by the enzyme. In this study, 13 bioactive herbal constituents were investigated for their inhibitory effects on UGT2B7 in HLM and RLM. Table 1 summarizes the effect of the constituents on UGT isoform activities in the literature. Based on the table, the in vitro inhibition of arecaidine, arecoline, (+)-catechin, gallic acid, kaempferol-3-rutinoside, quercetin, vanillin, vitexin, isovitexin and zerumbone on UGT2B7 has not been reported. Meanwhile, the effects of andrographolide, mangiferin and mitragynine against the activity of UGT2B7 have been previously screened in vitro. However, no study on andrographolide’s effect in RLM has been published. There is also no reported data on the effect of mangiferin and mitragynine on UGT2B7 in both HLM and RLM.

ZDV was used as the probe substrate to determine the UGT2B7 isoform activity in the current study. ZDV glucuronide formed was analyzed using a modified and validated high performance liquid chromatography (HPLC) method. Meanwhile, human and rat liver microsomes were used to investigate interspecies differences, as rats are the most common animals utilized pre-clinical in vivo studies. Data generated in this study will provide useful information for researchers with an intention to carry out further in vivo study in rats and humans as well as to provide a better understanding of the risks associated with the concomitant use of herbal medicines with pharmaceutical drugs among the general public including healthcare professionals.

## 2. Results

### 2.1. HPLC Method Validation

An HPLC method to quantify zidovudine glucuronide (ZDVG) in liver microsomes was validated in terms of specificity, linearity, sensitivity, precision, accuracy, extraction recovery and matrix effect according to the US Food and Drug Administration (FDA) [41] and International Conference on Harmonisation (ICH) [42].

#### 2.1.1. Specificity

**Figure 1:**
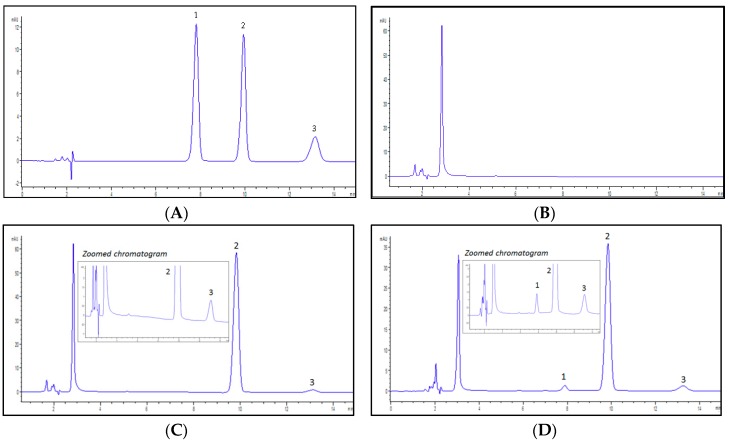
Representative chromatograms detected at λ = 267 nm. (**A**) a solution containing a mixture of zidovudine glucuronide (ZDVG), zidovudine (ZDV) and internal standard, 4-methylumbelliferone glucuronide (4MUG) at concentration of 150 μM. ZDVG, ZDV and 4MUG were eluted at 7.9, 9.9 and 13.1 min, respectively; (**B**) a HLM incubation matrix solution. No peaks were detected; (**C**) a HLM incubation assay in the absence of UDPGA. No metabolite (ZDVG) peak was detected whilst 4MUG was eluted at 13.1 min; (**D**) a HLM incubation assay in the presence of UDPGA. ZDVG and 4MUG were detected at 7.9 min and 13.1 min. (1) ZDVG; (2) ZDV; (3) 4MUG.

The peaks of ZDVG, ZDV and the internal standard, 4-methylumbelliferone glucuronide (4MUG), eluted at retention times of 7.9, 9.9 and 13.1 min, respectively, without overlapping. The specificity of this proposed analytical method was determined by the absence of endogenous interferences at the retention times of peaks of interest which were ZDVG and 4MUG. The chromatograms in Figure 1 show that there were no interferences from the endogenous substances on the retention times of both peaks.

#### 2.1.2. Linearity and Sensitivity

The calibration curve of the ratio of ZDVG/4MUG peak areas against ZDVG concentration was linear, with the regression equation y = 0.0811x − 0.0098 and a correlation coefficient of 0.9998. Meanwhile, the limits of detection (LOD) and quantification (LOQ) of ZDVG were 0.5 μM and 1.5 μM respectively.

#### 2.1.3. Precision and Accuracy

The precision and accuracy for intra- and inter-day of the method were determined with LOD and quality control (QC) samples of ZDVG at concentrations of 1.5, 4.5, 12.5 and 22.5 µM. The data is tabulated in Table 2. 

The intra-day precision ranged from 0.78% to 2.18% and the accuracy ranged from −4.39% to 8.58%. For inter-day assessment, precision ranged from 1.68% to 2.96% while accuracy ranged from −1.03% to 7.94%. All observed data were less than ±15% for QC samples and ±20% for the LOQ sample as suggested by the FDA [41].

#### 2.1.4. Extraction Recovery and Matrix Effect

Determinations of extraction recovery and matrix effect were conducted to measure the effectiveness of the sample preparation to generate values close to the actual sample values without any extraction and matrix influences. QC samples were used to evaluate both factors. The extraction recovery of ZDVG in HLM incubation assay after centrifugation was 101.25%, 98.55% and 97.69% for the low, medium and high QC samples, respectively. No significant matrix effect towards ZDVG was observed as well. Recovery of ZDVG from matrix effect was more than 96% for all QC samples. Table 3 shows the recovery obtained for both factors.

### 2.2. Zidovudine (ZDV) Glucuronidation in HLM

#### 2.2.1. Optimization of HLM and RLM Incubation Condition

**Figure 2:**
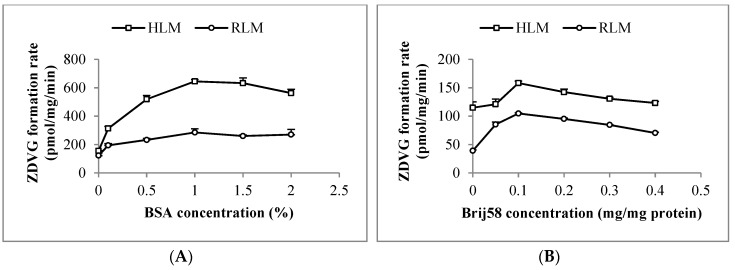
Optimization of (**A**) bovine serum albumin (BSA) and (**B**) polyethylene glycol hexadecyl ether (Brij58) concentration in incubation with human (HLM) and rat liver microsomes (RLM). Each point represents the mean of zidovudine glucuronides (ZDVG) formation rate ± standard deviation (SD) for triplicates (*n* = 3).

Latency of UGT2B7 enzyme activity in HLM and RLM was removed using 0.1 mg/mg of polyethylene glycol hexadecyl ether (Brij58). Following that, an addition of 1% (*w*/*v*) of bovine serum albumin (BSA) into the HLM and RLM incubation mixtures increased ZDVG formation by 4-fold and 2.5-fold, respectively, compared to the one without BSA (Figure 2). Moreover, formation of ZDVG with HLM was linear with protein concentration (up to 0.5 mg/mL) at an incubation time of 120 min and linear with time (up to 180 min) at a HLM concentration of 0.2 mg/mL. Meanwhile, formation of ZDVG with RLM was linear with protein concentration (up to 6 mg/mL) at an incubation time of 90 min and incubation time (up to 180 min) at RLM concentration of 2 mg/mL.

#### 2.2.2. ZDV Glucuronidation Kinetics in HLM and RLM

**Figure 3:**
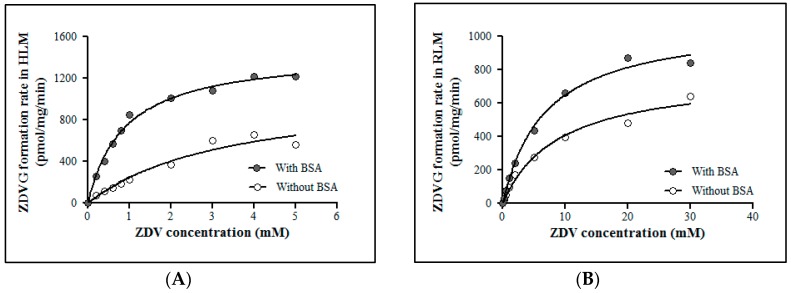
Michaelis-Menten plots for zidovudine (ZDV) glucuronidation catalyzed by (**A**) human liver microsomes (HLM) and (**B**) rat liver microsomes (RLM) with and without 1% (*w*/*v*) of bovine serum abumin (BSA). Each point represents the mean of zidovudine glucuronides (ZDVG) formation rate ± standard deviation (SD) for triplicates (*n* = 3).

ZDV glucuronidation kinetics in HLM and RLM were evaluated in the absence and presence of 1% (*w*/*v*) BSA (Figure 3). The addition of 1% (*w*/*v*) BSA in HLM and RLM incubation assays did not affect the maximal velocity (V_max_) value significantly. However, the Michaelis constant (K_m_) value was decreased approximately 4-fold and 2.5-fold for HLM and RLM incubation assays respectively compared to the assays without BSA. This condition results in a corresponding increase in the intrinsic clearance (CL_int_) of ZDV in both HLM and RLM. These kinetic data are shown in Table 4.

#### 2.2.3. Inhibition Studies of Herbal Bioactive Constituents on ZDV Glucuronidation in HLM

**Figure 4:**
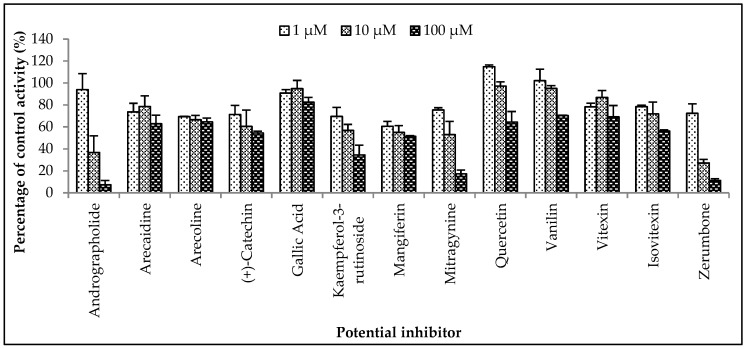
The screening of herbal bioactive constituents on zidovudine (ZDV) glucuronidation in human liver microsomes (HLM). Panels above show concentrations of the inhibitors used. Each bar represents the mean percentage activity relative to control ± standard deviation (SD) for triplicates (*n* = 3).

**Figure 5:**
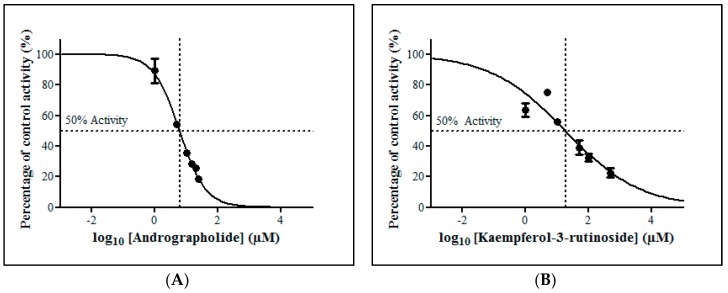

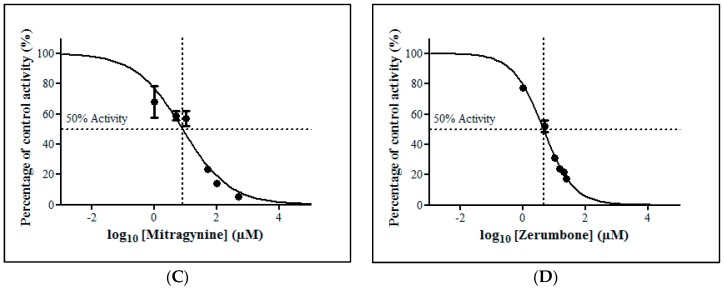
The inhibitory effect of (**A**) andrographolide, (**B**) kaempferol-3-rutinoside, (**C**) mitragynine and (**D**) zerumbone on zidovudine (ZDV) glucuronidation in human liver microsomes (HLM). Each point represents the mean percentage of relative ZDV-UGT2B7 specific activity over control ± standard deviation (SD) for triplicates (*n* = 3). Data points were fitted to non-linear regression (curve fit) using GraphPad Prism 5.01 to determine an IC_50_ value. Goodness of fit *R*^2^ values were greater than 0.9.

The inhibitory potential of the herbal constituents on ZDV glucuronidation was evaluated using three different concentrations for each constituent which were 1 µM, 10 µM and 100 µM. Among them, andrographolide, kaempferol-3-rutinoside, mitragynine and zerumbone had shown 50% inhibition at concentration below 100 µM (Figure 4). These four constituents were then further evaluated to determine their precise half-maximal inhibitory concentration (IC_50_) using additional concentration points. The IC_50_s obtained for andrographolide, kaempferol-3-rutinoside, mitragynine and zerumbone were 6.18 ± 1.27 μM, 18.56 ± 8.62 μM, 8.11 ± 4.48 μM and 4.57 ± 0.23 μM, respectively. The IC_50_ plots are illustrated in Figure 5.

#### 2.2.4. Inhibition Studies of Herbal Bioactive Constituents on ZDV Glucuronidation in RLM

**Figure 6:**
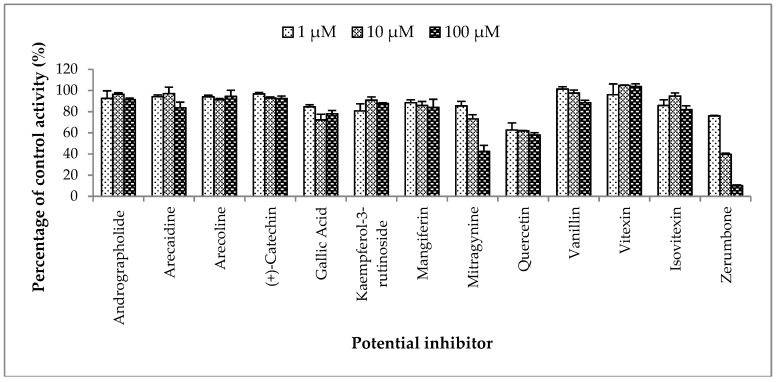
The screening of herbal bioactive constituents on zidovudine (ZDV) glucuronidation in rat liver microsomes (RLM). Panels above show concentrations of the inhibitors used. Each bar represents the mean percentage activity relative to control ± standard deviation (SD) for triplicates (*n* = 3).

**Figure 7:**
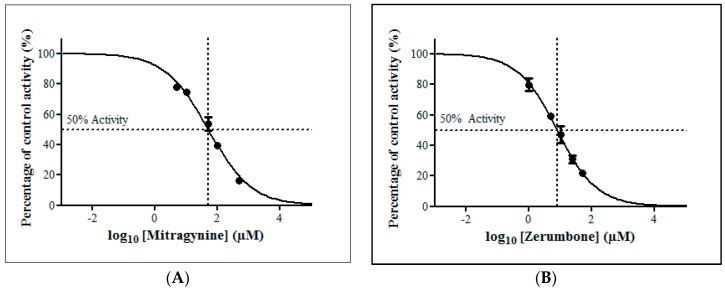
The inhibitory effect of (**A**) mitragynine and (**B**) zerumbone on zidovudine (ZDV) glucuronidation in rat liver microsomes (RLM). Each point represents the mean percentage of relative ZDV-UGT2B7 specific activity over control ± standard deviation (SD) for triplicates (*n* = 3). Data points were fitted to non-linear regression (curve fit) using GraphPad Prism 5.01 to determine an IC_50_ value. Goodness of fit *R*^2^ values were greater than 0.9.

The inhibitory potential of the thirteen herbal bioactive constituents on ZDV glucuronidation in RLM was evaluated similarly as in HLM. In RLM, only mitragynine and zerumbone had inhibited ZDV glucuronidation higher than 50% at concentration below 100 µM (Figure 6). The result of precise IC_50_ values show that mitragynine and zerumbone exhibited inhibition on ZDV glucuronidation in RLM with IC_50_ values of 51.20 ± 5.95 µM and 8.14 ± 2.12 µM respectively. The IC_50_ plots for both constituents are shown in Figure 7.

## 3. Discussion

An optimized and validated HPLC-UV method was successfully applied to the determination of ZDV glucuronidation activity in liver microsomes. Thirteen herbal bioactive constituents including andrographolide, arecaidine, arecoline, (+)-catechin, gallic acid, kaempferol-3-rutinoside, mangiferin, mitragynine, quercetin, vanillin, vitexin, isovitexin and zerumbone were studied to determine their inhibitory potential on ZDV glucuronidation in HLM and RLM.

Prior to the inhibition study, an optimized ZDV incubation assay in HLM and RLM had been established under a steady state condition where the metabolites formed were not more than 30% formed [43] and were linearly correlated with protein concentration and incubation time. Meeting these requirements allows the straightforward data interpretation as the reversible reaction between the substrate and the product formed will not take place [44]. Latency of UGT2B7 enzyme in microsomes also had been removed using an optimum Brij58 concentration. In addition, an addition of 1% (*w*/*v*) BSA content into the incubation mixture of HLM and RLM also had reduced the K_m_ value of ZDV without significantly affecting its V_max_ value as shown in Table 3. V_max_ is the maximal velocity of the enzymatic reaction whilst K_m_ or Michaelis constant is the substrate concentration at half of V_max_. The reduction of K_m_ value is consistent with previous studies [45,46] although we had used a lower BSA content. It is postulated that BSA sequesters the fatty acids released during the course of microsomal incubation in which these fatty acids have been reported to be inhibitors of UGT2B7 enzyme [46].

K_m_ and V_max_ values with inclusion of 1% of BSA for HLM are 0.88 ± 0.05 mM and 1450 ± 26.06 pmol/mg/min, respectively, whereas for RLM, they are 6.77 ± 0.60 mM and 1089 ± 33.50 pmol/mg/min, respectively. The K_m_ value in RLM is almost 8-fold higher when compared to HLM. However, the V_max_ value of ZDV glucuronidation in RLM is not significantly different from the one obtained in HLM. This trend is much easier to see when the liver microsomal intrinsic clearance (CL_int_) of ZDV glucuronidation is determined in both HLM and RLM. Fundamentally, CL_int_ is defined as the ability of the liver to metabolize the drug in the absence of flow limitations and binding to cells or proteins in the blood. The in vitro CL_int_ may be derived from the enzymes kinetic data which is V_max_/K_m_ [47,48]. Therefore, the CL_int_ in HLM is 1.65 µL/mg/min whereas in RLM it is 0.16 µL/mg/min. There is a 10-fold difference of CL_int_ value between HLM and RLM. Furthermore, it has been reported that approximately 75% and 10% of a ZDV dose were excreted as glucuronides in humans [49] and rats [50], respectively. Consequently, the in vivo systemic clearance of ZDV in humans was estimated to be 1.01 L/h/kg for 70 kg of man [51] which is almost 6-fold greater than the systemic clearance in rats which is 0.18 L/h/kg for 0.25 kg of rat [50,52]. Thus, it might be concluded that the capability of rats to metabolize ZDV is weaker compared to humans.

The inhibition study in HLM showed that four out of thirteen herbal bioactive constituents (andrographolide, kaempferol-3-rutinoside, mitragynine and zerumbone) inhibited ZDV glucuronidation activity. Andrographolide is a major constituent isolated from the herbal plant *Andrographis paniculata* (Burm. f.). A literature survey shows a wide spectrum of pharmacological significance of *A. paniculata* and andrographolide, including hepatoprotective, hypoglycaemic, cardioprotective, anti-inflammatory, immune stimulatory, anti-cancer, anti-HIV and antimalarial activities [53,54]. Andrographolide was found to inhibit UGT2B7 enzyme activity in previous studies [19,20,22,23]. Ma et al., reported that andrographolide non-competitively inhibited ZDV glucuronidation in HLM with an inhibition constant (K_i_) value of 6.1 µM [23]. As IC_50_ = K_i_ for non-competitive inhibition mechanism [55], therefore, the IC_50_ of their study can be deduced to be equal to the K_i_ value. Our study found a comparable IC_50_ value of 6.18 ± 1.27 µM. However in our previous study, andrographolide had shown weak inhibition in HLM when 4-methylumbelliferone (4MU) was used as a substrate [24]. This could be because 4MU is a nonspecific UGT substrate which is mainly conjugated by UGT1A6 in humans and rats [56], unlike ZDV which is a specific and selective substrate of UGT2B7 [57]. This selectivity factor might be the reason for the differences in the observed results.

In order to interpret herb-drug interactions in vivo, the in vitro IC_50_ value obtained should be viewed as the in vivo concentration [27]. Therefore, the in vivo inhibition potential of andrographolide in human liver can be estimated by comparing its IC_50_ value with its bioavailability in human plasma. A previous study [58] showed that the maximum plasma concentration of andrographolide in humans after oral administration of 20 mg of andrographolide four times a day was 1.34 µg/mL (3.8 µM). The value is lower than the IC_50_ value of 6.18 µM obtained in this study. Another two pharmacokinetic studies of andrographolide showed that its maximum concentration in plasma were 58.6 ng/mL (0.17 µM) and 32.41 ng/mL (0.09 µM) after ingestion of 200 mg and 32.64 mg of andrographolide for three times a day, respectively [59,60]. As the andrographolide concentration in plasma reportedly ranged from 0.09 to 3.8 µM which is less than the obtained IC_50_ value in HLM (6.18 ± 1.27 µM), inhibition of hepatic systemic clearance of UGT2B7 substrates in humans seems implausible.

In spite of that, due to first pass metabolism, orally administered drugs may be metabolized by the small intestine prior to their entrance into the liver [61]. Based on estimates of intestinal volume that range from approximately 0.5 to 5 L anticipating a 100% bioavailability, minor protein binding and minor accumulation of the substances [62], the intestinal concentration of the herbal constituents after their ingestion can also be predicted and compared with the hepatic IC_50_ value obtained in this study. Thus, following consumption of 20 to 200 mg of andrographolide, the expected intestinal concentration of andrographolide is 4 μg/mL (11.4 μM) and 400 μg/mL (1141 μM). These concentrations are higher than the IC_50_ for UGT2B7 inhibition by andrographolide in HLM. Thus, it is postulated that inhibition of intestinal glucuronidation of UGT2B7 substrates by andrographolide may be possible. Furthermore, UGT2B7 is also expressed in the human small intestine [63].

This study has also shown mitragynine to have a strong inhibition towards ZDV glucuronidation in HLM with an IC_50_ value of 8.11 ± 4.48 μM. This IC_50_ value is lower than the IC_50_ value obtained previously with 4MU in HLM. Previously, mitragynine at a concentration of 1 μM inhibited 4MU glucuronidation in HLM by approximately 30% of control activity with an IC_50_ value higher than 1000 μM [37]. This difference might be due to the non-specificity of 4MU as a substrate on UGT isoforms as aforementioned. Mitragynine may be a specific UGT2B7 inhibitor, however, further study on its inhibition mechanism needs to be carried out for confirmation.

Briefly, mitragynine is a major constituent of *Mitragyna speciosa*, a herbal plant with opium-like effects and coca-like simulative ability [64,65]. Due to this, *M. speciosa* has been widely taken to alleviate opioid withdrawal symptoms [66,67]. In addition, *M. speciosa* is also commonly consumed in combination with other drugs of abuse such as codeine or diphenhydramine cough syrup, amphetamine, ketamine, antidepressants drugs and opioids to achieve an intense feeling of pleasure [68,69]. Opioids such as codeine, morphine and oxycodone are known to be metabolized by UGT2B7 [5] whereas ketamine had been shown to inhibit UGT2B7 in vitro [37,70].

Reported concentrations of mitragynine in postmortem blood samples ranged from 230 μg/L (0.58 µM) [71] to 1006 μg/L (2.52 µM) [72]. Meanwhile, a study conducted in humans showed that the maximum plasma concentration of mitragynine was 0.105 µg/mL (0.26 µM) following the ingestion of a *M. speciosa* tea containing 11 mg mitragynine for seven days followed by 23 mg of mitragynine on the eighth day [73]. Since the plasma level of mitragynine is less than the observed IC_50_ of mitragynine obtained in this study which is 8.11 ± 4.48 µM, it is predicted that inhibition of systemic clearance of UGT2B7 substrates by mitragynine is unlikely to occur. In contrast, intestinal concentrations of mitragynine are expected to fall in the range of 4.6 mg/L (11.5 µM) to 553 mg/L (1388 µM) following the consumption of *M. speciosa* juice/tea containing 23 to 276.5 mg mitragynine [73,74]. Therefore, mitragynine may inhibit the intestinal glucuronidation of UGT2B7 substrates when they are concomitantly consumed.

Kaempferol-3-rutinoside is a naturally derived flavonoid that is found in almost all herbal, as well as in vegetables and fruits. Flavonoids have been reported to possess a broad spectrum of health benefits due to their antioxidative, anti-inflammatory, anti-allergic, anti-mutagenic and anticancer properties, which are widely utilized in a variety of nutraceutical, pharmaceutical, medicinal and cosmetic applications [75]. Similar to andrographolide and mitragynine, kaempferol-3-rutinoside did not significantly inhibit UGT enzymes in HLM when 4MU was used as a substrate [24]. On the other hand, it had inhibited UGT2B7-catalyzed glucuronidation of ZDV in HLM with an IC_50_ value of 18.56 ± 8.62 µM in this study. Since the magnitude is modest, herb-drug interaction involving this constituent is unlikely to occur. Furthermore, there is no data of kaempferol-3-rutinoside plasma concentration in humans to be compared with the obtained IC_50_ value. The daily recommended allowance (RDA) of kaempferol-3-rutinoside is also unavailable to estimate its intestinal concentration.

**Figure 8:**
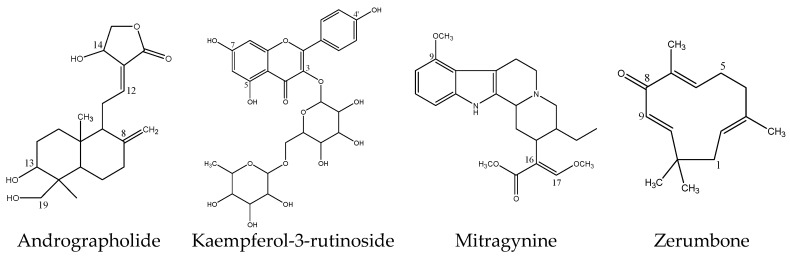
Chemical structures of inhibitory herbal bioactive constituents on UGT2B7 enzyme.

Zerumbone is a bioactive compound which is found in many species of the Zingiberaceae family such as *Zingiber zerumbet*, *Zingiber amaricans* and *Zingiber aromaticum*, although most abundantly in *Z. zerumbet* [76]. Zerumbone has been described to be a promising drug for the treatment of different types of cancer such as colon, breast, cervix and liver cancer [76]. There are limited studies regarding zerumbone metabolism and pharmacokinetics. Zerumbone does not have the common nucleophilic functional groups like aliphatic alcohols, phenols, carboxylic acids, thiols or aromatic and aliphatic amines to form glucuronides [77,78]. However, a C-linked glucuronide can be formed at its acidic carbon atom (C-9 as shown in Figure 8). Moreover, glucuronidation of zerumbone may also take place on its carbonyl group in which the group is tautomerized into an enol group prior to the conjugation which consequently produces the zerumbone *O*-glucuronide [79].

In terms of its effect on drug metabolizing enzyme activity, zerumbone had been shown to induce the Phase II gluthathione-S-transferases enzyme [80] and moderately inhibit metabolism mediated by CYP3A4 [81] in vitro. However, there is no published data for zerumbone effect on UGT enzymes. This study may be the first to report on zerumbone potently inhibiting ZDV glucuronidation in HLM with an IC_50_ value of 4.57 ± 0.23 µM. Unfortunately, zerumbone concentration in human plasma and its RDA were unavailable to be compared with the IC_50_ value obtained.

The inhibitory potency of the thirteen constituents investigated in HLM was also studied in RLM. Overall, the inhibition trend in RLM was similar to the trend in HLM for most constituents, only differing in their magnitude. Out of the four herbal constituents that had strongly inhibited ZDV glucuronidation in HLM, only two showed a similar magnitude of inhibition in RLM. These constituents were mitragynine and zerumbone, with IC_50_ values of 51.20 ± 5.95 µM and 8.14 ± 2.12 µM, respectively. The only constituent that showed a different trend in this study was andrographolide which strongly inhibited ZDV glucuronidation in HLM but showed weak inhibition in RLM. This may be due to the orthologous nature of the UGT2B7 enzyme. It is known that UGT2B1, an orthologous enzyme to UGT2B7 is expressed in rats instead of UGT2B7 itself. UGT2B1 does not always catalyze the same compounds as UGT2B7 [3] even though it has been described to catalyze the same classes of substances as UGT2B7 [82].

To the best of our knowledge, no study of andrographolide inhibition on UGT enzymes specifically UGT2B7 in RLM has been published. Nonetheless, a recent study conducted by Tian et al. described that when andrographolide was used as a substrate, it was glucuronidated in HLM but not in RLM [83].

## 4. Materials and Methods

### 4.1. Chemical and Reagents

3′-Azido-3′-deoxythymidine (ZDV), 4-MU β-d-glucuronide hydrate (4MUG), andrographolide, arecaidine hydrochloride, arecoline hydrobromide, diclofenac (sodium salt), kaempferol-3-*O*-rutinoside, gallic acid, quercetin, mangiferin, zerumbone, bovine serum albumin (BSA), polyethylene glycol hexadecyl ether (Brij58), uridine 5′-diphosphoglucuronic acid trisodium salt (UDPGA), tris(hydroxymethyl)aminomethane hydrochloride (Tris-HCl) and magnesium chloride (MgCl_2_) and pooled human liver microsomes (Product No. M0567) were purchased from Sigma-Aldrich (St. Louis, MO, USA). 3′-Azido-3′-deoxythymidine β-d-glucuronide sodium salt (ZDVG) was purchased from Santa Cruz Biotechnology (Dallas, TX, USA). (+)-Catechin hydrate and mitragynine were purchased from Chromadex (Irvine, CA, USA). Vitexin and isovitexin were obtained from Carl Roth GmbH (Karlsruhe, Germany). Vanillin was purchased from BDH chemicals Ltd. (Poole, UK). Potassium chloride (KCl) was obtained from Bendosen Laboratory Chemicals (Selangor, Malaysia). Sodium carbonate (Na_2_CO_3_), sodium hydroxide (NaOH), glycerol and potassium dihydrogen phosphate (KH_2_PO_4_) were obtained from Systerm^®^ (Shah Alam, Malaysia). Dipotassium hydrogen phosphate (K_2_HPO_4_) was obtained from Riedel-de Hӓen (Seelze, Germany). Folin and Ciocalteu’s reagent and triethylamine were obtained from R&M Marketing (Essex, UK). Acetonitrile (HPLC grade) and absolute ethanol (analytical grade) were obtained from RCl Labscan Limited (Bangkok, Thailand). Dimethyl sulfoxide (DMSO) and formic acid HPLC grade (99.5%) were obtained from Fisher Scientific (Loughborough, UK).

### 4.2. Animals

Six male Sprague Dawley rats (150–200 g) were obtained from Animal House, Universiti Sains Malaysia (USM). The rats were placed under 12 h light and 12 h dark conditions with controlled temperature (25 ± 2 °C) for seven days before the start of the experiments. Water and food *ad libitium* were provided to the rats. The animals were maintained and handled according to the recommendations of the USM ethical committee which approved the animal experiments with reference number of USM/Animal Ethics Approval/2013/(90) (527). The livers were taken out from the rats after sacrificing them by cervical dislocation for microsomes preparation.

### 4.3. Rat Liver Microsomes Preparation

The livers were rinsed promptly with ice-cold distilled water followed by ice-cooled 67 mM potassium phosphate buffer (pH 7.4) containing 1.15% (*w*/*v*) KCl after removal from the rats. The livers were then homogenized in three volumes of the same buffer using Potter-Elvehjem homogenizer after blotted dry and weighed. After that, the homogenate was centrifuged at 12,500× *g* for 20 min at 4 °C. The resultant supernatant was further centrifuged at 100,000× *g* for 60 min at 4 °C to obtain the microsomal pellets. Each pellet obtained was scrapped and reconstituted in 0.3 mL of 67 mM potassium phosphate buffer with 1.15% (*w*/*v*) KCl and 20% (*v*/*v*) glycerol before homogenizing them together to obtain pooled microsomes. Microsomes were then transferred into several microfuge tubes and stored at −80 °C until used.

### 4.4. Instrumentation and Chromatographic Conditions

HPLC was performed using a Jasco HPLC system with intelligent UV/Vis detector (Jasco International Co., Ltd., Tokyo, Japan) fitted with a reversed phase Hypersil Gold C18 column (150 × 4.6 mm I.D., 5 µm particle size; Thermo Fisher Scientific, Waltham, MA, USA). The optimized method used the isocratic mobile phase consisting of 0.5% formic acid in deionized water and acetonitrile (90:10%; *v*/*v*) which ran at 1.0 mL/min for 15 min. A sample of 10 µL injection volume was monitored at 267 nm wavelength.

### 4.5. Validation of HPLC Method for Quantification of ZDVG

The HPLC method was validated for specificity, linearity, sensitivity, precision, accuracy, extraction recovery and matrix effect according to the guidelines issued by FDA [41] and International Conference on Harmonisation (ICH) [42].

#### 4.5.1. Specificity

Specificity was determined by analyzing a HLM incubation sample to evaluate for any endogenous interferences at retention times of ZDVG and 4MUG (IS). A comparison study was conducted on chromatograms of a blank HLM incubation matrix sample, HLM incubation sample without UDPGA, and HLM incubation sample with UDPGA. Blank sample spiked with pure standards of ZDVG, ZDV and 4MUG served as reference.

#### 4.5.2. Linearity

The linearity of the developed HPLC method was evaluated by a calibration curve constructed using six different concentrations of ZDVG at 1.5, 3, 5, 10, 15, and 30 µM spiked in incubation buffer. The reading for each concentration was taken for three different days with five replicates per day. A total of fifteen replicates (*n* = 15) for each concentration were used to construct the curve. The peak area ratios of ZDVG and 4MUG (IS) were plotted against the nominal concentrations of ZDVG spiked in incubation buffer.

#### 4.5.3. Sensitivity

Sensitivity of HPLC method was evaluated by determining the limits of detection (LOD) and quantification (LOQ) using data generated from the calibration curve. LOD and LOQ were calculated as LOD = 3.3 (SD_y-int_/S) and LOQ = 10 (SD_y-int_/S). SD_y-int_ is standard deviation of y-intercept and S is the slope of the calibration curve.

#### 4.5.4. Precision and Accuracy

Precision and accuracy were determined using LOQ and quality control (QC) samples at concentrations 1.5, 4.5, 12.5 and 22.5 μM of ZDVG spiked in incubation buffer. Intra- and inter-day precision and accuracy were assessed by analyzing LOQ and QC samples in five replicates on the same day and over three consecutive days, respectively. Precision was expressed as percent coefficient of variance (% CV) whilst accuracy was expressed as percent relative mean error (% RME). CV (%) and RME (%) were calculated as follows: CV (%) = (standard deviation/mean) × 100; RME (%) = ([measured concentration − nominal concentration]/[nominal concentration]) × 100.

#### 4.5.5. Extraction Recovery and Matrix Effect

Five replicates of QC samples were used to evaluate extraction recovery of ZDVG and matrix effect on ZDVG. Extraction recovery was calculated by comparing the analytical response of ZDVG spiked pre-centrifugation to the one spiked post-centrifugation. The matrix effect was evaluated by comparing the analytical response of ZDVG spiked post-centrifugation to the one spiked directly into distilled water. In this experiment, the analytical response measured was the mean of peak area ratio of ZDVG/4MUG.

### 4.6. Microsomal Incubation Assay

An incubation mixture in a total volume of 200 µL contained 100 mM Tris-HCl buffer at pH 7.4, 5 mM MgCl_2_, microsomal protein (0.2 mg/mL for HLM assay and 2 mg/mL for RLM assay) and 0.1 mg/mg protein of Brij58 was kept in ice for 10 min. After that, ZDV (concentration at or below the K_m_ value) and 1% (*w*/*v*) of bovine serum albumin (BSA) were added into the incubation mixture. The incubation volume was made up to 200 µL with distilled water in a 1.5 mL polypropylene microcentrifuge tube. After pre-incubation at 37 °C for 10 min in a shaking water bath, a reaction was started by the addition of 5 mM UDPGA and was further incubated (120 min for HLM and 90 min for RLM). Following the incubation, the reaction was terminated by adding 100 µL ice-cold acetonitrile containing 75 µM of 4MUG as the internal standard (IS). The sample was allowed for protein precipitation on ice for 15 min, then vortexed shortly, and centrifuged at 15,000 rpm for 10 min at 4 °C. Supernatant was transferred into HPLC vials for HPLC analysis.

### 4.7. Optimization of Incubation Conditions

Preliminary assays in terms of optimization of Brij58 concentration, BSA content and linearity of microsomal protein concentration as well as incubation time were performed to ensure ZDV glucuronidation experiments were carried out under initial linear velocity condition (product formation < 30%). Brij58 concentration was optimized at concentration ranged from 0–0.4 mg/mg of protein. Optimization of BSA concentration ranged from 0–0.2% (*w*/*v*) in the presence of optimized Brij58 concentration in the incubation assay was then carried out. After that, the linearity of protein concentration and incubation time with ZDVG formation was assessed with the inclusion of the optimized concentration of Brij58 and BSA.

### 4.8. Enzyme Kinetics of ZDV Glucuronidation

Once the initial velocity conditions had been established, the ZDV concentrations were varied to generate a saturation curve for determination of Michaelis constant (K_m_) and maximal velocity of reaction (V_max_) values of ZDV glucuronidation in HLM and RLM. The experiments were conducted in the absence and presence of BSA. Under optimized conditions, substrate concentrations used in incubation assay were varied from 0–5 mM of ZDV for HLM assay and 0–30 mM of ZDV for RLM assay. All assays were performed in triplicates (*n* = 3). The enzyme kinetic parameters (K_m_ and V_max_) were obtained by fitting kinetic models to the experimental data using GraphPad Prism version 5.01 for Windows.

### 4.9. Preparation of Potential Inhibitors Solution

Stock solutions for the herbal constituents were prepared at concentrations of 50 mM by dissolving them in their respective solvents. Andrographolide, arecoline, arecaidine, mangiferin and vanillin were dissolved in distilled water. (+)-Catechin, mitragynine and gallic acid were dissolved in ethanol. Meanwhile, kaempferol-3-rutinoside, quercetin, vitexin, isovitexin and zerumbone were dissolved in dimethyl sulfoxide (DMSO). Working solutions were prepared at concentrations of 5 mM in distilled water so that the final organic solvent concentrations in incubations were less than 2% (*v*/*v*).

### 4.10. Inhibition Study of ZDV Glucuronidation in HLM and RLM

Thirteen herbal constituents were screened for inhibition of ZDV glucuronidation in HLM and RLM. Screening experiments using three concentrations (1 µM, 10 µM and 100 µM) of each herbal constituent were conducted to screen for the inhibitory potential of the constituent. All incubation assays were performed in triplicate (*n* = 3). Constituents that showed at least 50% inhibition on ZDV glucuronidation at concentrations below 100 µM, was further studied using several concentrations to obtain the precise IC_50_ value.

### 4.11. Data Analysis

The ZDVG concentration formed was quantified using the calibration curve of ZDVG. Final UGT2B7 specific activity value was expressed in pmol/min/mg protein. However, UGT2B7 specific activity was reported as the percentage of control activity (%) by comparing the UGT2B7 specific activity in the presence of the inhibitor to the one without inhibitor (control). IC_50_ value point where the enzyme activity was reduced by half was found graphically on the basis of the plot created using GraphPad Prism 5.0.1 for Windows (GraphPad Software, La Jolla, CA, USA) under dose–response inhibition (log [inhibitor] vs. normalized response -- variable slope) model. K_m_ and V_max_ were determined using the same software under the enzyme-kinetic inhibition (substrate inhibition) model.

## 5. Conclusions

In summary, our study of the inhibitory potential of thirteen herbal bioactive constituents showed that andrographolide, kaemperol-3-rutinoside, mitragynine and zerumbone inhibit ZDV glucuronidation in HLM whilst only mitragynine and zerumbone inhibited ZDV glucuronidation in RLM. Hence, herb-drug interactions are a possibility if drugs that are highly metabolized by UGT2B7 are taken concomitantly with andrographolide, kaempferol-3-rutinoside, mitragynine and zerumbone. Herb-herb interactions may also occur if a herbal medicine consisting of a mixture of the herbal constituents is consumed. However, in order to confirm this finding, a clinical study needs to be conducted. In addition, due to the differences in the inhibitory outcomes in HLM and RLM, data for UGT2B7 inhibition in rats need to be extrapolated with caution to humans.

## Figures and Tables

**Table 1:** The effect of selected herbal constituents on UDP-glucuronosyltransferase (UGT) isoforms activities.

	Herbal Constituent	In Vitro Study	In Vivo/Ex Vivo Study	References
1.	Andrographolide	UGT nonspecific 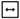 (HLM)	UGT nonspecific ↑ (rats)	[19,20,21,22,23,24]
		UGT1A3 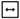 (supersomes)		
		UGT1A4 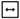 (supersomes)		
		UGT1A6 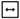 (supersomes)		
		UGT1A7 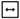 (supersomes)		
		UGT1A8 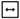 (supersomes)		
		UGT1A9 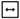 (supersomes)		
		UGT1A10 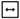 (supersomes)		
		UGT2B4 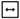 (supersomes)		
		UGT2B7 ↓ (HLM, supersomes)		
2.	Arecaidine	-	-	
3.	Arecoline	UGT nonspecific 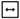 (RLM)	-	[25]
4.	(+)-Catechin	-	-	
5.	Kaempferol	UGT nonspecific ↓ (HLM)	UGT1A1 ↑ (mice)	[26,27,28,29,30]
		UGT nonspecific ↑ (mammalian cells)		
		UGT1A1 ↓ (supersomes)		
		UGT1A1 ↑ (mammalian cells)		
		UGT1A1 ↑ (mammalian cells)		
		UGT1A3 ↓ (supersomes)		
		UGT1A6 ↓ (supersomes)		
		UGT1A9 ↓ (HLM, HIM, supersomes)		
		UGT2B7 ↓ (supersomes)		
		UGT2B10 ↓ (supersomes)		
	Kaempferol-3-rutinoside	UGT nonspecific 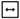 (HLM)		[24]
6.	Gallic acid	UGT2B17 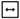 (supersomes)	-	[31]
7.	Mangiferin	UGT1A1 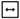 (hepatocytes)	-	[32,33,34]
		UGT1A3 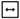 (supersomes)		
		UGT1A6 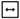 (supersomes)		
		UGT1A7 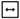 (supersomes)		
		UGT1A8 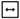 (supersomes)		
		UGT1A9 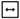 (hepatocytes, supersomes)		
		UGT1A10 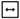 (supersomes)		
		UGT2B4 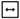 (supersomes)		
		UGT2B7 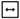 (hepatocytes, supersomes)		
		UGT2B15 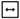 (supersomes)		
		UGT2B17 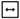 (supersomes)		
8.	Mitragynine	UGT nonspecific 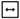 (HLM, RLM)	UGT nonspecific ↑ (rats)	[35,36,37]
		UGT1A1 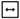 (supersomes)		
		UGT2B7 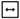 (supersomes)		
9.	Quercetin	UGT nonspecific ↑ (mammalian cells)	UGT nonspecific ↑ (rat)	[26,27,29,31,38,39]
		UGT1A1 ↑ (mammalian cells)		
		UGT1A1 ↓ (supersomes)		
		UGT1A3 ↓ (supersomes)		
		UGT1A4 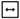 (supersomes)		
		UGT1A6 ↑ (mammalian cells)		
		UGT1A6 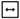 (supersomes)		
		UGT1A9 ↓ (HLM, RLM, supersomes)		
		UGT2B10 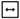 (supersomes)		
		UGT2B17 ↓ (supersomes)		
10.	Vanillin	UGT nonspecific ↓	-	[40]
11.	Vitexin	UGT nonspecific 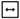 (HLM)	-	[24]
12.	Isovitexin	UGT nonspecific 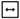 (HLM)	-	[24]
13.	Zerumbone	-	-	-

↓, inhibition of enzyme activity; ↑, activation of enzyme activity; 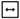, weak inhibition or no effect on enzyme activity; HLM, human liver microsomes; RLM, rat liver microsomes.

**Table 2:** Intra- and inter-day precision (% CV) and accuracy (% RME) of zidovudine glucuronides (ZDVG). Measurements were taken for five replicates per day for three days.

Calibrator (ZDVG)	Concentration (μM)		CV (%)	RME (%)
	Nominal	Measured		
Intra-day (*n* = 5)				
LOQ	1.5	1.63	2.18	8.56
QC1	4.5	4.55	1.67	1.06
QC2	12.5	12.23	2.27	−2.17
QC3	22.5	21.51	0.78	−4.39
Inter-day (*n* = 15)				
LOQ	1.5	1.62	1.68	7.94
QC1	4.5	4.59	1.60	2.06
QC2	12.5	12.37	2.84	−1.03
QC3	22.5	22.29	2.96	−0.92

LOQ = limit of quantification; QC = quality control; CV = coefficient of variance; RME = relative mean error.

**Table 3:** Extraction recovery and matrix effect of zidovudine glucuronides (ZDVG). Measurement was made from five independent experiments (*n* = 5).

ZDVG Calibrators	QC1	QC2	QC3
	1.5 µM	12.5 µM	12.5 µM
ZDVG spiked sample ^1^			
In incubation matrix pre-centrifugation	0.3697	0.9717	1.7869
In incubation matrix post-centrifugation	0.3651	0.9860	1.8291
In distilled water	0.3665	1.0250	1.7996
Extraction recovery (%)	101.25	98.55	97.69
Matrix Effect (%)	99.64	96.19	101.64

^1^ Data represents the mean peak area ratio of ZDVG/4MUG; QC = quality control.

**Table 4:** Derived kinetic constants for zidovudine glucuronidation in human (HLM) and rat liver microsomes (RLM) obtained from the incubation with and without 1% (*w*/*v*) bovine serum albumin (BSA). Data are presented as mean ± SD.

	Without 1% BSA			With 1% BSA		
	V_max_ (pmol/mg/min)	K_m_ (mM)	CL_int_ (µL/mg/min)	V_max_ (pmol/mg/min)	K_m_ (mM)	CL_int_ (µL/mg/min)
**HLM**	1092 ± 19.10	3.40 ± 0.70	0.32	1450 ± 26.05	0.88 ± 0.05	1.65
**RLM**	770 ± 39.82	8.84 ± 1.19	0.09	1089 ± 33.50	6.77 ± 0.60	0.16

V_max_ = maximal velocity; K_m_ = Michaelis constant; CL_int_ = intrinsic clearance.

## References

[1] Meech R., Mackenzie P.I. (1997). Structure and function of uridine diphosphate glucuronosyltransferases. Clin. Exp. Pharmacol. Physiol..

[2] Wells P.G., Mackenzie P.I., Chowdhury J.R., Guillemette C., Gregory P.A., Ishii Y., Hansen A.J., Kessler F.K., Kim P.M., Chowdhury N.R. (2004). Glucuronidation and the UDP-glucuronosyltransferases in health and disease. Drug Metab. Dispos..

[3] King C., Rios G., Green M., Tephly T. (2000). UDP-glucuronosyltransferases. Curr. Drug Metab..

[4] Coffman B.L., Rios G.R., King C.D., Tephly T.R. (1997). Human UGT2B7 catalyzes morphine glucuronidation. Drug Metab. Dispos..

[5] Middleton R.K., Helms R.A., Quan D.J., Herfindal E.T., Gourley D.R. (2006). Drug Interactions. Textbook of Therapeutics: Drug and Disease Management.

[6] Mano Y., Usui T., Kamimura H. (2007). Inhibitory potential of nonsteroidal anti-inflammatory drugs on UDP-glucuronosyltransferase 2B7 in human liver microsomes. Eur. J. Clin. Pharmacol..

[7] Jin C., Miners J., Lillywhite K., Mackenzie P. (1993). Complementary deoxyribonucleic acid cloning and expression of a human liver uridine diphosphate-glucuronosyltransferase glucuronidating carboxylic acid-containing drugs. J. Pharmacol. Exp. Therap..

[8] Barbier O., Turgeon D., Girard C., Green M.D., Tephly T.R., Hum D.W., Bélanger A. (2000). 3′-azido-3′-deoxythimidine (AZT) is glucuronidated by human UDP-glucuronosyltransferase 2B7 (UGT2B7). Drug Metab. Dispos..

[9] Jin C.J., Miners J., Burchell B., Mackenzie P. (1993). The glucuronidation of hydroxylated metabolites of benzo [α] pyrene and 2-acetylaminofluorene by cDNA-expressed human UDP-glucuronosyltransferases. Carcinogenesis.

[10] Chang T.K., Chen J., Yeung E.Y. (2006). Effect of Ginkgo biloba extract on procarcinogen-bioactivating human CYP1 enzymes: Identification of isorhamnetin, kaempferol, and quercetin as potent inhibitors of CYP1B1. Toxicol. Appl. Pharmacol..

[11] Damery S., Gratus C., Grieve R., Warmington S., Jones J., Routledge P., Greenfield S., Dowswell G., Sherriff J., Wilson S. (2011). The use of herbal medicines by people with cancer: A cross-sectional survey. Br. J. Cancer.

[12] Bahall M. (2017). Prevalence, patterns, and perceived value of complementary and alternative medicine among HIV patients: A descriptive study. BMC Complement. Altern. Med..

[13] Manya K., Champion B., Dunning T. (2012). The use of complementary and alternative medicine among people living with diabetes in Sydney. BMC Complement. Altern. Med..

[14] Rivera J., Loya A., Ceballos R. (2013). Use of herbal medicines and implications for conventional drug therapy medical sciences. Altern. Integr. Med..

[15] You B.H., Gong E.C., Choi Y.H. (2018). Inhibitory effect of sauchinone on UDP-glucuronosyltransferase (UGT) 2B7 activity. Molecules.

[16] Rachlis A., Fanning M.M. (1993). Zidovudine toxicity. Drug Saf..

[17] Dutta B. (2015). Study of secondary metabolite constituents and curcumin contents of six different species of genus Curcuma. J. Med. Plants.

[18] Salleh N.A.M. (2015). Effects of *Curcuma Xanthorrhiza Roxb.* on Phase II Drug Metabolizing Enzymes. Master’s Thesis.

[19] Uchaipichat V. (2018). In vitro inhibitory effects of major bioactive constituents of *Andrographis paniculata*, *Curcuma longa* and *Silybum marianum* on human liver microsomal morphine glucuronidation: A prediction of potential herb-drug interactions arising from andrographolide, curcumin and silybin inhibition in humans. Drug Metab. Pharmacokinet..

[20] Ismail S., Hanapi N.A., Abd Halim M.R., Uchaipichat V., Mackenzie P. (2010). Effects of *Andrographis paniculata* and *Orthosiphon stamineus* extracts on the glucuronidation of 4-Methylumbelliferone in human UGT isoforms. Molecules.

[21] Chen H.W., Huang C.S., Liu P.F., Li C.C., Chen C.T., Liu C.T., Chiang J.R., Yao H.T., Lii C.K. (2013). *Andrographis paniculata* extract and andrographolide modulate the hepatic drug metabolism system and plasma tolbutamide concentrations in rats. Evid.-Based Complement. Altern. Med..

[22] Abidin S.Z., Liew W., Ismail S., Chan K., Mahmud R. (2014). Inhibitory effects of active constituents and extracts of *Andrographis paniculata* on UGT1A1, UGT1A4, and UGT2B7 enzyme activities. Int. J. Pharm. Pharm. Sci..

[23] Ma H.Y., Sun D.X., Cao Y.F., Ai C.Z., Qu Y.Q., Hu C.M., Jiang C., Dong P.P., Sun X.Y., Hong M. (2014). Herb-drug interaction prediction based on the high specific inhibition of andrographolide derivatives towards UDP-glucuronosyltransferase (UGT) 2B7. Toxicol. Appl. Pharmacol..

[24] Husni Z., Ismail S., Zulkiffli M.H., Afandi A., Haron M. (2017). In vitro inhibitory effects of *Andrographis paniculata*, *Gynura procumbens*, *Ficus deltoidea*, and *Curcuma xanthorrhiza* extracts and constituents on human liver glucuronidation activity. Pharmacogn. Mag..

[25] Grancharov K., Engelberg H., Naydenova Z., Müller G., Rettenmeier A.W., Golovinsky E. (2001). Inhibition of UDP-glucuronosyltransferases in rat liver microsomes by natural mutagens and carcinogens. Arch. Toxicol..

[26] Saracino M.R., Lampe J.W. (2007). Phytochemical regulation of UDP-glucuronosyltransferases: Implications for cancer prevention. Nutr. Cancer.

[27] Mohamed M.E.F., Frye R.F. (2010). Inhibition of intestinal and hepatic glucuronidation of mycophenolic acid by *Ginkgo biloba* extract and flavonoids. Drug Metab. Dispos..

[28] Gufford B.T., Chen G., Lazarus P., Graf T.N., Oberlies N.H., Paine M.F. (2014). Identification of diet-derived constituents as potent inhibitors of intestinal glucuronidation. Drug Metab. Dispos..

[29] Schwab N., Skopp G. (2014). Identification and preliminary characterization of UDP-glucuronosyltransferases catalyzing formation of ethyl glucuronide. Anal. Bioanal. Chem..

[30] Tsai M.S., Wang Y.H., Lai Y.Y., Tsou H.K., Liou G.G., Ko J.L., Wang S.H. (2018). Kaempferol protects against propacetamol-induced acute liver injury through CYP2E1 inactivation, UGT1A1 activation, and attenuation of oxidative stress, inflammation and apoptosis in mice. Toxicol. Lett..

[31] Jenkinson C., Petroczi A., Naughton D.P. (2012). Red wine and component flavonoids inhibit UGT2B17 in vitro. Nutr. Rev..

[32] Liu H., Wang K., Tang Y., Sun Z., Jian L., Li Z., Wu B., Huang C. (2011). Structure elucidation of in vivo and in vitro metabolites of mangiferin. J. Pharm. Biomed. Anal..

[33] Rodeiro I., José Gómez-Lechón M., Perez G., Hernandez I., Herrera J.A., Delgado R., Castell J.V., Teresa D.M. (2013). *Mangifera indica* L. extract and mangiferin modulate Cytochrome P450 and UDP-Glucuronosyltransferase enzymes in primary cultures of human hepatocytes. Phytother. Res..

[34] Sun D., Zhang C.Z., Ran R.X., Cao Y.F., Du Z., Fu Z.W., Huang C.T., Zhao Z.Y., Zhang W.H., Fang Z.Z. (2017). In vitro comparative study of the inhibitory effects of mangiferin and its aglycone norathyriol towards UDP-glucuronosyl transferase (UGT) isoforms. Molecules.

[35] Azizi J., Ismail S., Mansor S.M. (2013). *Mitragyna speciosa* Korth leaves extracts induced the CYP450 catalyzed aminopyrine-N-demethylase (APND) and UDP-glucuronosyl transferase (UGT) activities in male Sprague-Dawley rat livers. Drug Metabol. Drug Interact..

[36] Xiao R., Wang J., Chen J., Sun L., Chen Y. (2014). Effects of arecoline on hepatic Cytochrome P450 activity and oxidative stress. J. Toxicol. Sci..

[37] Haron M., Ismail S. (2015). Effects of mitragynine and 7-hydroxymitragynine (the alkaloids of *Mitragyna speciosa* Korth) on 4-methylumbelliferone glucuronidation in rat and human liver microsomes and recombinant human uridine 5’-diphospho-glucuronosyltransferase isoforms. Pharmacogn. Res..

[38] Bock K.W., Eckle T., Ouzzine M., Fournel-Gigleux S. (2000). Coordinate induction by antioxidants of UDP-glucuronosyltransferase UGT1A6 and the apical conjugate export pump MRP2 (multidrug resistance protein 2) in Caco-2 cells. Biochem. Pharmacol..

[39] Van der Logt E., Roelofs H., Nagengast F., Peters W. (2003). Induction of rat hepatic and intestinal UDP-glucuronosyltransferases by naturally occurring dietary anticarcinogens. Carcinogenesis.

[40] Salleh N.M., Ismail S., Ibrahim M.N.M. (2017). The inhibition of hepatic and renal glucuronidation of p-Nitrophenol and 4-methylumbelliferone by oil palm empty fruit bunch lignin and its main oxidation compounds. Pharmacogn. Mag..

[41] Food and Drug Administration (FDA) (2001). Guidance for Industry: Bioanalytical Method Validation.

[42] ICH Harmonised Tripartite Guidelines (2005). Validation of Analytical Procedures: Text and Methodology Q2 (R1).

[43] Food and Drug Administration (FDA) (2006). Guidance for Industry: Drug Interaction Studies: Study Design, Data Analysis, Implications for Dosing, and Labeling Recommendations.

[44] Cornish-Bowden A. (2013). The origins of enzyme kinetics. FEBS Lett..

[45] Uchaipichat V., Winner L.K., Mackenzie P.I., Elliot D.J., Williams J.A., Miners J.O. (2006). Quantitative prediction of in vivo inhibitory interactions involving glucuronidated drugs from in vitro data: The effect of fluconazole on zidovudine glucuronidation. Br. J. Clin. Pharmacol..

[46] Rowland A., Gaganis P., Elliot D.J., Mackenzie P.I., Knights K.M., Miners J.O. (2007). Binding of inhibitory fatty acids is responsible for the enhancement of UDP-glucuronosyltransferase 2B7 activity by albumin: Implications for in vitro-in vivo extrapolation. J. Pharmacol. Exp. Ther..

[47] Houston J.B. (1994). Utility of in vitro drug metabolism data in predicting in vivo metabolic clearance. Biochem. Pharmacol..

[48] Griffin S.J., Houston J.B. (2004). Comparison of fresh and cryopreserved rat hepatocyte suspensions for the prediction of in vitro intrinsic clearance. Drug Metab. Dispos..

[49] Blum M., Liao S., Good S. (1988). Pharmacokinetics and bioavailability of zidovudine in humans. Am. J. Med..

[50] Mays D.C., Dixon K.F., Balboa A., Pawluk L.J., Bauer M.R., Nawoot S., Gerber N. (1991). A nonprimate animal model applicable to zidovudine pharmacokinetics in humans: Inhibition of glucuronidation and renal excretion of zidovudine by probenecid in rats. J. Pharmacol. Exp. Therap..

[51] Boase S., Miners J.O. (2002). In vitro*–in vivo* correlations for drugs eliminated by glucuronidation: Investigations with the model substrate zidovudine. Br. J. Clin. Pharmacol..

[52] Wannachaiyasit S., Chanvorachote P., Nimmannit U. (2008). A novel anti-HIV dextrin–zidovudine conjugate improving the pharmacokinetics of zidovudine in rats. AAPS PharmSciTech.

[53] Jarukamjorn K., Nemoto N. (2008). Pharmacological aspects of *Andrographis paniculata on* health and its major diterpenoid constituent andrographolide. J. Health Sci..

[54] Akbar S. (2011). *Andrographis paniculata*: A review of pharmacological activities and clinical effects. Altern. Med. Rev..

[55] Brandt R.B., Laux J.E., Yates S.W. (1987). Calculation of inhibitor K_i_ and inhibitor type from the concentration of inhibitor for 50% inhibition for Michaelis-Menten enzymes. Biochem. Med. Metab. Biol..

[56] Hanioka N., Jinno H., Tanaka-Kagawa T., Nishimura T., Ando M. (2001). Determination of UDP-glucuronosyltransferase UGT1A6 activity in human and rat liver microsomes by HPLC with UV detection. J. Pharm. Biomed. Anal..

[57] Court M.H., Krishnaswamy S., Hao Q., Duan S.X., Patten C.J., von Moltke L.L., Greenblatt D.J. (2003). Evaluation of 3′-azido-3′-deoxythymidine, morphine, and codeine as probe substrates for UDP-glucuronosyltransferase 2B7 (UGT2B7) in human liver microsomes: Specificity and influence of the UGT2B7*2 polymorphism. Drug Metab. Dispos..

[58] Panossian A., Hovhannisyan A., Mamikonyan G., Abrahamian H., Hambardzumyan E., Gabrielian E., Goukasova G., Wikman G., Wagner H. (2000). Pharmacokinetic and oral bioavailability of andrographolide from *Andrographis paniculata* fixed combination Kan Jang in rats and human. Phytomedicine.

[59] Xu L., Xiao D.-W., Lou S., Zou J.-J., Zhu Y.-B., Fan H.-W., Wang G.-J. (2009). A simple and sensitive HPLC–ESI-MS/MS method for the determination of andrographolide in human plasma. J. Chromatogr. B.

[60] Pholphana N., Panomvana D., Rangkadilok N., Suriyo T., Ungtrakul T., Pongpun W., Thaeopattha S., Satayavivad J. (2016). A simple and sensitive LC-MS/MS method for determination of four major active diterpenoids from *Andrographis paniculata* in human plasma and its application to a pilot study. Planta Med..

[61] Lin J.H., Chiba M., Baillie T.A. (1999). Is the role of the small intestine in first-pass metabolism overemphasized?. Pharmacol. Rev..

[62] Hellum B.H., Hu Z., Nilsen O.G. (2007). The induction of CYP1A2, CYP2D6 and CYP3A4 by six trade herbal products in cultured primary human hepatocytes. Basic Clin. Pharmacol. Toxicol..

[63] Turgeon D., Carrier J.-S., Lévesque E., Hum D.W., Bélanger A. (2001). Relative enzymatic activity, protein stability, and tissue distribution of human steroid-metabolizing UGT2B subfamily members. Endocrinology.

[64] Chan K., Pakiam C., Rahim R.A. (2007). Psychoactive plant abuse: The identification of mitragynine in ketum and in ketum preparations. Bulletin of Narcotics.

[65] Babu K.M., McCurdy C.R., Boyer E.W. (2008). Opioid receptors and legal highs: *Salvia divinorum* and Kratom. Clinic. Toxicol..

[66] Boyer E.W., Babu K.M., Adkins J.E., McCurdy C.R., Halpern J.H. (2008). Self-treatment of opioid withdrawal using kratom (*Mitragynia speciosa* korth). Addiction.

[67] Vicknasingam B., Narayanan S., Beng G.T., Mansor S.M. (2010). The informal use of ketum (*Mitragyna speciosa*) for opioid withdrawal in the northern states of peninsular Malaysia and implications for drug substitution therapy. Int. J. Drug Policy.

[68] Tungtananuwat W., Lawanprasert S. (2010). Fatal 4x100: Homemade kratom juice cocktail. J. Health Res..

[69] Likhitsathian S., Jiraporncharoen W., Aramrattana A., Angkurawaranon C., Srisurapanont M., Thaikla K., Assanangkornchai S., Kanato M., Perngparn U., Jarubenja R. (2018). Polydrug use among kratom users: Findings from the 2011 Thailand National Household Survey. J. Subst. Use.

[70] Uchaipichat V., Raungrut P., Chau N., Janchawee B., Evans A.M., Miners J.O. (2011). Effects of ketamine on human UDP-glucuronosyltransferases in vitro predict potential drug-drug interactions arising from ketamine inhibition of codeine and morphine glucuronidation. Drug Metab. Dispos..

[71] McIntyre I.M., Trochta A., Stolberg S., Campman S.C. (2014). Mitragynine ‘Kratom’ related fatality: A case report with postmortem concentrations. J. Anal. Toxicol..

[72] Karinen R., Fosen J.T., Rogde S., Vindenes V. (2014). An accidental poisoning with mitragynine. Forensic Sci. Int..

[73] Trakulsrichai S., Sathirakul K., Auparakkitanon S., Krongvorakul J., Sueajai J., Noumjad N., Sukasem C., Wananukul W. (2015). Pharmacokinetics of mitragynine in man. Drug Des. Dev. Ther..

[74] Singh D., Müller C.P., Vicknasingam B.K. (2014). Kratom (*Mitragyna speciosa*) dependence, withdrawal symptoms and craving in regular users. Drug Alcohol Depend..

[75] Panche A., Diwan A., Chandra S. (2016). Flavonoids: An overview. J. Nutr. Sci..

[76] Rahman H.S., Rasedee A., Yeap S.K., Othman H.H., Chartrand M.S., Namvar F., Abdul A.B., How C.W. (2014). Biomedical properties of a natural dietary plant metabolite, zerumbone, in cancer therapy and chemoprevention trials. Biomed. Res. Int..

[77] Sorich M.J., McKinnon R.A., Miners J.O., Smith P.A. (2006). The importance of local chemical structure for chemical metabolism by human uridine 5′-diphosphate- glucuronosyltransferase. J. Chem. Inf. Model..

[78] Rowland A., Miners J.O., Mackenzie P.I. (2013). The UDP-glucuronosyltransferases: Their role in drug metabolism and detoxification. Int. J. Biochem. Cell Biol..

[79] Powers R.H., Dean D.E. (2015). Biotransformation. Forensic Toxicology Mechanism and Pathology.

[80] Nakamura Y., Yoshida C., Murakami A., Ohigashi H., Osawa T., Uchida K. (2004). Zerumbone, a tropical ginger sesquiterpene, activates phase II drug metabolizing enzymes. FEBS Lett..

[81] Usia T., Iwata H., Hiratsuka A., Watabe T., Kadota S., Tezuka Y. (2004). Sesquiterpenes and flavonol glycosides from *Zingiber aromaticum* and their CYP3A4 and CYP2D6 inhibitory activities. J. Nat. Prod..

[82] Mackenzie P.I., Owens I.S., Burchell B., Bock K.W., Bairoch A., Bélanger A., Fournel-Gigleux S., Green M., Hum D.W., Iyanagi T. (1997). The UDP glycosyltransferase gene superfamily: Recommended nomenclature update based on evolutionary divergence. Pharmacogenetics.

[83] Tian X., Liang S., Wang C., Wu B., Ge G., Deng S., Liu K., Yang L., Ma X. (2015). Regioselective glucuronidation of andrographolide and its major derivatives: Metabolite identification, isozyme contribution, and species differences. AAPS J..

